# Supplementary material for: Diagnostic Utility of Bronchoalveolar Lavage Flow Cytometric Leukocyte Profiling in Interstitial Lung Disease and Infection
Source: Biomolecules. 2025 Apr 17;15(4):597. doi: 10.3390/biom15040597 (PMC12025232; doi:10.3390/biom15040597)

Supplementary Figure S1

BAL with normal leukocyte content

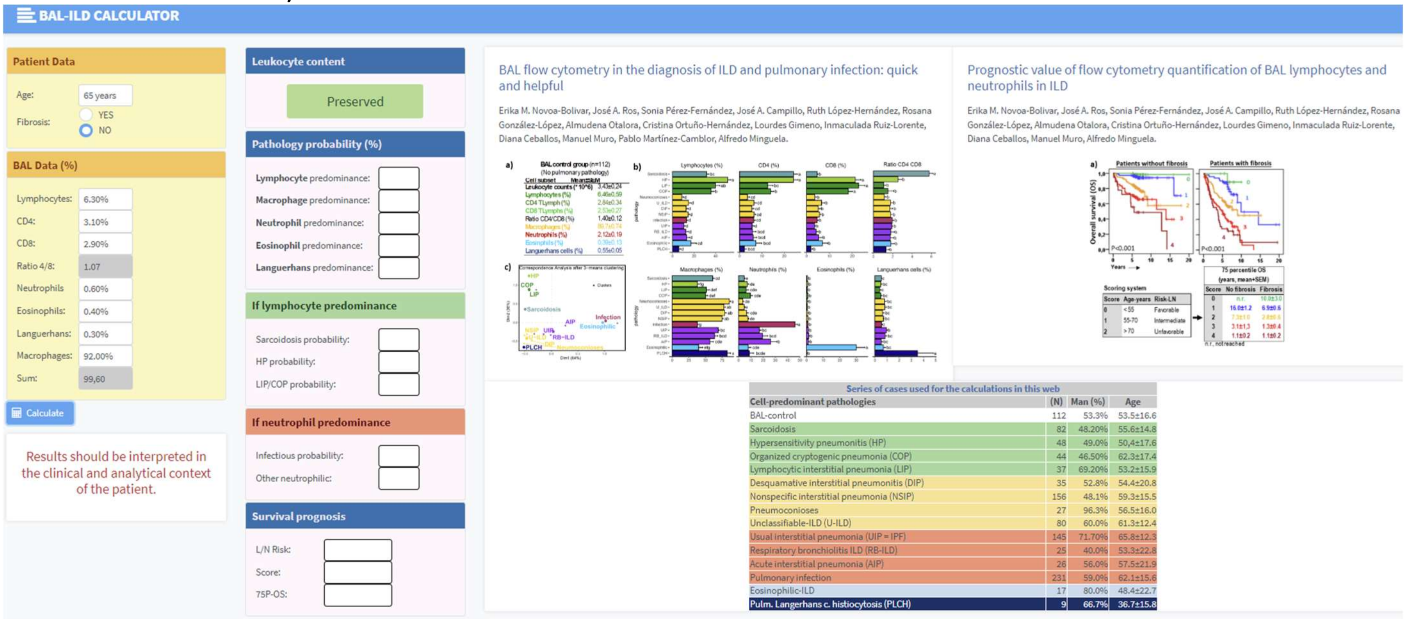

Cell-predominant pathologies	(N)	Man (%)	Age
BAL-control	112	53.3%	53.5±16.6
Sarcoidosis	82	48.20%	55.6±14.8
Hypersensitivity pneumonitis (HP)	48	49.0%	50,4±17.6
Organized cryptogenic pneumonia (COP)	44	46.50%	62.3±17.4
Lymphocytic interstitial pneumonia (LIP)	37	69.20%	53.2±15.9
Desquamative interstitial pneumonitis (DIP)	35	52.8%	54.4±20.8
Nonspecific interstitial pneumonia (NSIP)	156	48.1%	59.3±15.5
Pneumoconioses	27	96.3%	56.5±16.0
Unclassifiable-ILD (U-ILD)	80	60.0%	61.3±12.4
Usual interstitial pneumonia (UIP = IPF)	145	71.70%	65.8±12.3
Respiratory bronchiolitis ILD (RB-ILD)	25	40.0%	53.3±22.8
Acute interstitial pneumonia (AIP)	26	56.0%	57.5±21.9
Pulmonary infection	231	59.0%	62.1±15.6
Eosinophilic-ILD	17	80.0%	48.4±22.7
Pulm. Langerhans c. histiocytosis (PLCH)	9	66.7%	36.7±15.8

**Eosinophilic ILD**<br>(LN-Risk: Favorable)<br>No fibrosis.	**PLCH**<br>(LN-Risk: Intermediate)<br>No fibrosis.	**Macrophagic**<br>(LN-Risk: Favorable)<br>No fibrosis.
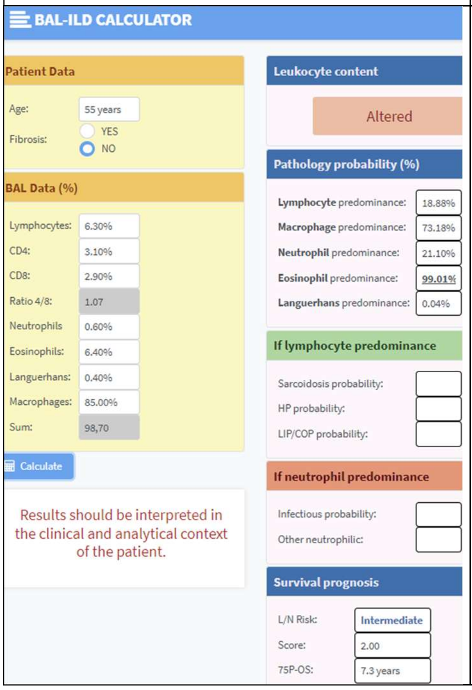	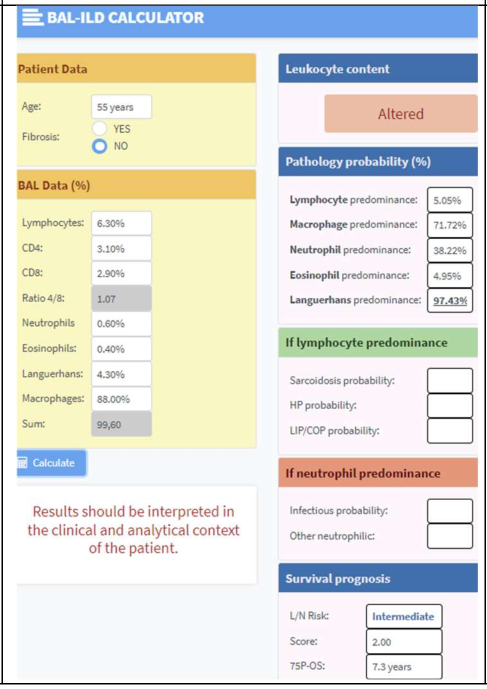	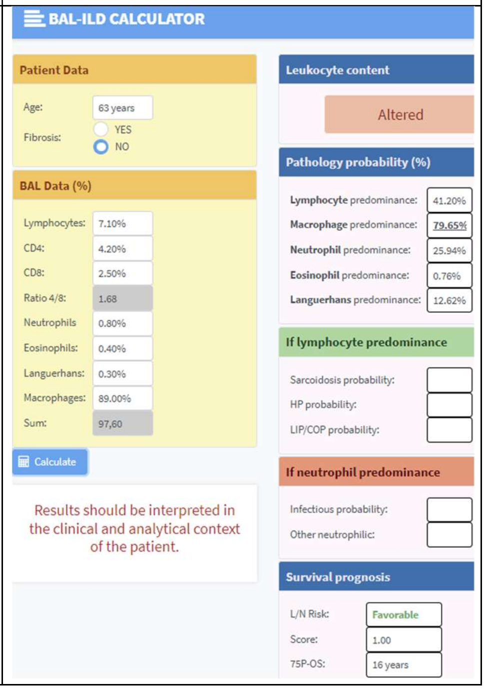

Lymphocytic (Sarcoidosis)
(LN-Risk: Favorable)
No fibrosis.

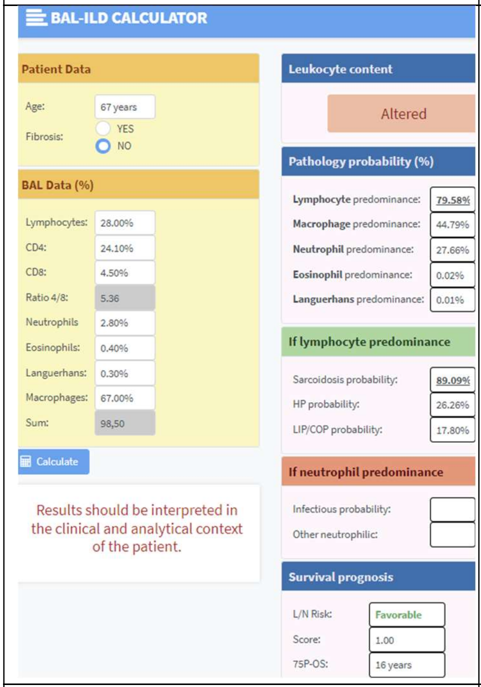

Lymphocytic (HP)
(LN-Risk: Intermediate)
With fibrosis.

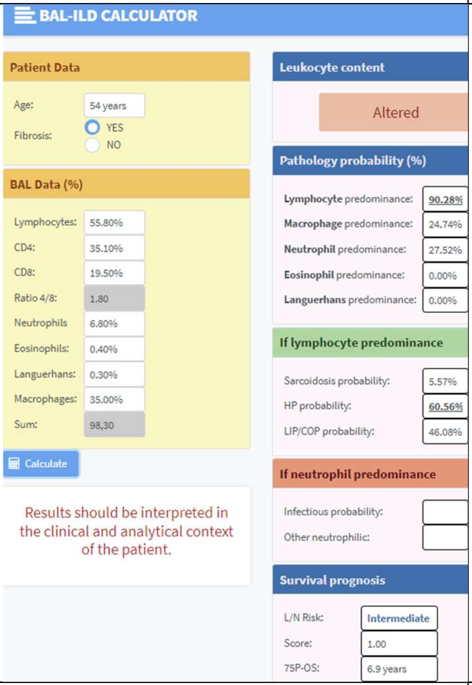

Lymphocytic (LIP or COP)
(LN-Risk: Favorable)
No fibrosis.

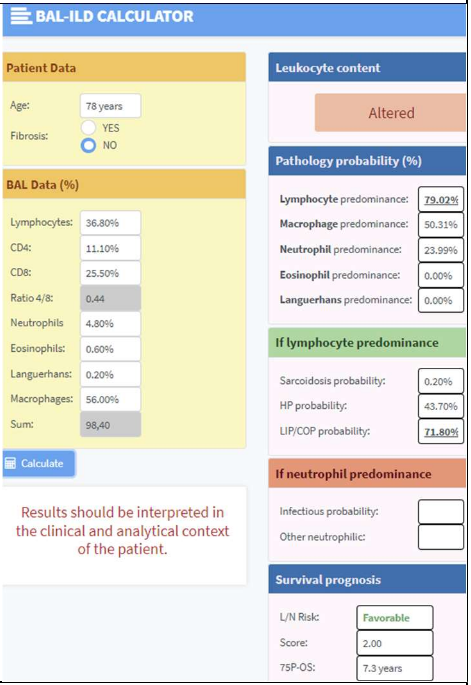

Neutrophilic (infectious)
(LN-Risk: Intermediate)
No fibrosis.

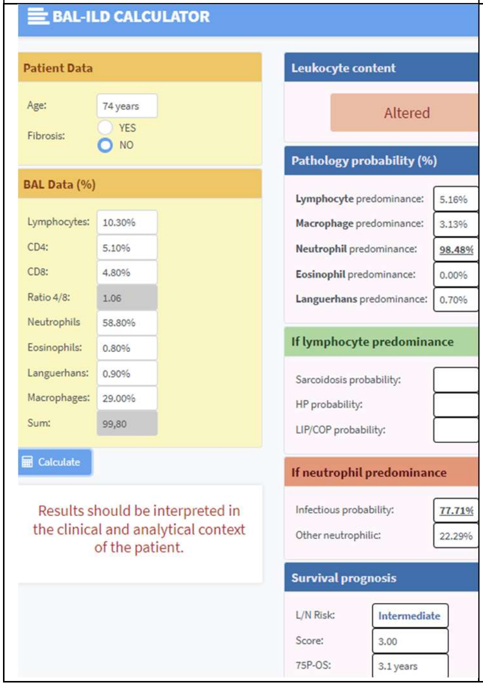

Neutrophilic (Other)
(LN-Risk: Unfavorable)
With fibrosis

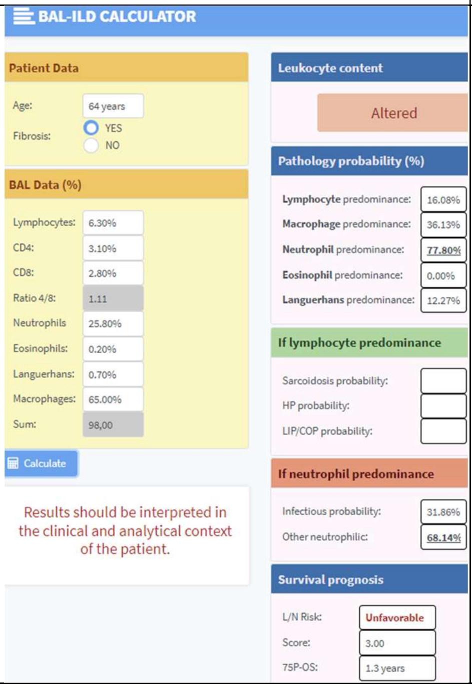

Supplement: Supplementary file 1 [file biomolecules-15-00597-s001.zip › Supplementary Figure S1.pdf]
