# Supplementary figures and images for: Diagnostic Utility of Bronchoalveolar Lavage Flow Cytometric Leukocyte Profiling in Interstitial Lung Disease and Infection
Source: Biomolecules. 2025 Apr 17;15(4):597. doi: 10.3390/biom15040597 (PMC12025232; doi:10.3390/biom15040597)

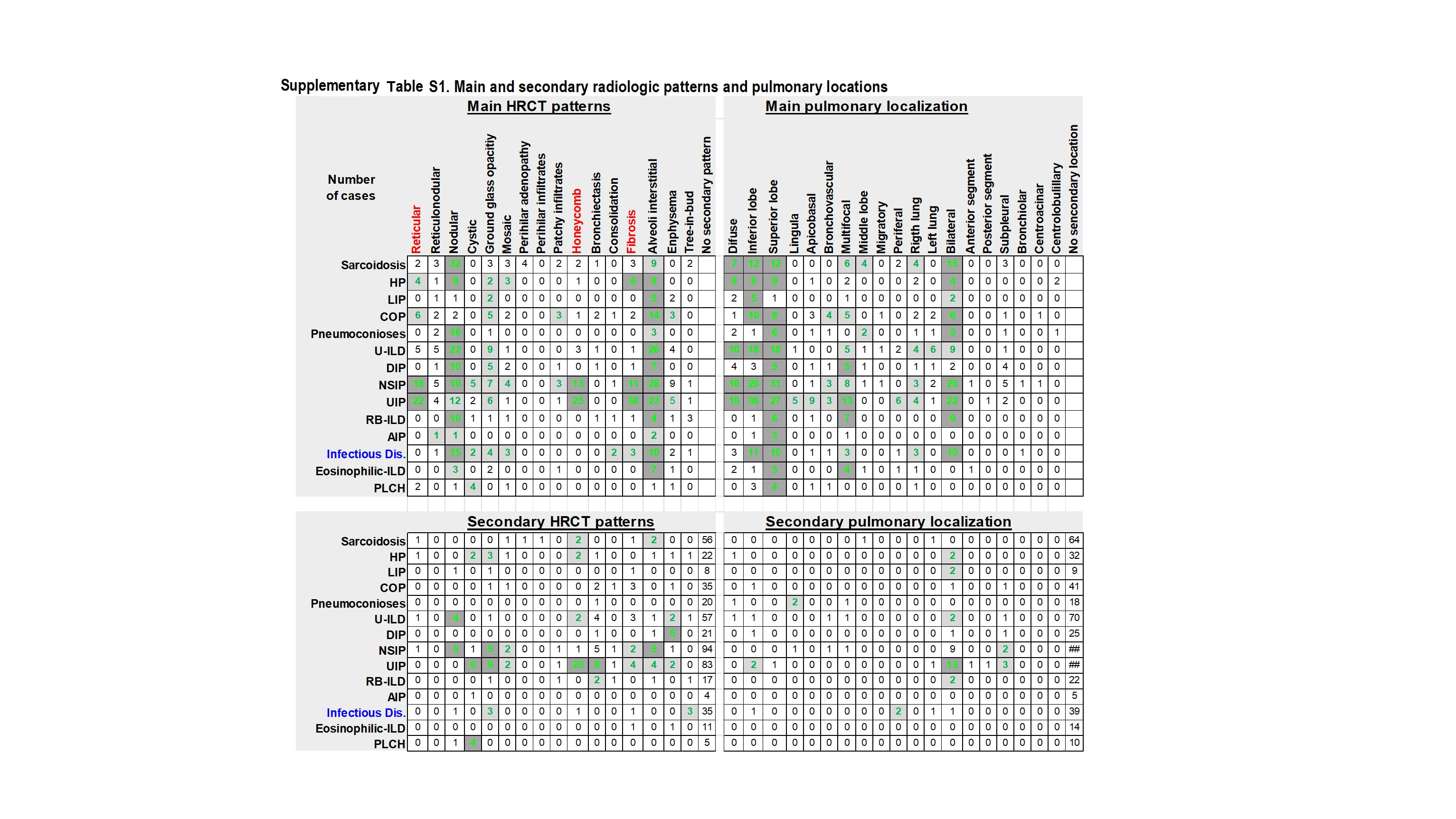

Supplement: Supplementary file 1 [file biomolecules-15-00597-s001.zip › Supplemenary Table S1.jpg]
